# Assessing strategies to mitigate the impacts of a pandemic in apparel supply chains

**DOI:** 10.1007/s12063-022-00345-w

**Published:** 2023-01-23

**Authors:** Naimur Rahman Chowdhury, Farhatul Janan, Priom Mahmud, Sharmine Akther Liza, Sanjoy Kumar Paul

**Affiliations:** 1 Department of Industrial and Systems Engineering, North Carolina State University, Raleigh, NC 27695 USA; 2 Department of Industrial Engineering, Clemson University, Clemson, SC 29634 USA; 3 Department of Systems and Industrial Engineering, University of Arizona, Tucson, AZ 85721 USA; 4 Department of Industrial and Systems Engineering, Lehigh University, Bethlehem, PA 18015 USA; 5 UTS Business School, University of Technology Sydney, Sydney, Australia

**Keywords:** COVID-19 pandemic, Supply chain, Mitigation strategies, Grey theory, Bi-level analytical network process

## Abstract

The COVID-19 pandemic has taught global businesses that a pandemic can put business dynamics in unforeseeable turbulence. The disruptions created by the pandemic in the apparel industry exposed the vulnerabilities of apparel supply chains (SCs). To recover the supply chain impacts (SCIs) during an unprecedented event such as the COVID-19 pandemic, apparel SCs need a robust framework that can identify, measure, and mitigate the severity of SCIs by assessing effective mitigation strategies. This study identifies 12 critical SCIs in apparel SCs during a pandemic and 17 mitigation strategies. To assess SCIs and mitigation strategies, a modified grey-based bi-level analytical network process (ANP) is proposed to deal with the complex relationship between the SCIs and mitigation strategies. A real-life case study is conducted from an apparel supply chain for validation purposes. The findings suggest that policymakers in apparel SCs should prioritize implementing government policies and financial aid to deal with increased material and operational costs, the sudden surge in the unemployment rate, cancellation of orders and delayed payment, and increased transportation costs during a pandemic. This study also contributes to the literature by providing a robust decision-making framework for practitioners to deal with the complexity of SCs during future pandemics.

## Introduction

The COVID-19 pandemic has shaken every aspect of human lives unanticipatedly (Chakraborty and Maity 2020). Consequently, global business and operations dynamics have been crippled and put under tremendous financial crises by the impacts of the pandemic (Chowdhury et al. 2020; Shrestha et al. 2020).

Apparel, one of the largest labor-intensive global manufacturing industries, has been at a severe stake due to enforced lockdowns, closing down of factories, and international distribution restrictions in the wake of the COVID-19 pandemic (Majumdar et al. 2020). Even though operations have recently been resumed, the impacts left by the pandemic have caused a massive drop in the economic performance of apparel SCs and put millions of workers in the sector at risk of unemployment (Kabir et al. 2021). Although the South Asian region has many large apparel exporters, it has not covered the financial loss it faced during the early phase of the COVID-19 pandemic due to the halt in procuring raw materials from the primary source, China (Islam et al. 2020a, b).

The impacts of the pandemic are even more prominent in emerging economies, such as Bangladesh, the second-largest apparel exporter. As it turned out, Bangladesh has seen a suspension or cancellation of orders worth USD 3.17 billion by June 2020 due to an unprecedented demand fall in the global market (Majumdar et al. 2020). It was predicted that Bangladesh might lose around $6 billion in export revenue amid order cancellations and factory shutdowns (Paul and Chowdhury 2020). Further, the unavailability of raw materials and restrictions on distribution also caused immense volatility in the local supply and demand of apparel SCs (Dohale et al. 2021a, b; Murmu et al. 2022). These impacts surely needed attention by the research community to recover the disrupted performance of the SC.

Realizing the need to mitigate the impacts of the pandemic, researchers have already focused on developing mitigation strategies for retrieving sustainable operations, economic restoration, and resilience of SCs in the context of the COVID-19 pandemic (Chowdhury et al. 2021b; Farooq et al. 2021; Mangano et al. 2022; Rahman et al. 2021; Rozhkov et al. 2022). Even though the studies present valuable insights into SCs during a pandemic on a global scale, there has been less focus on comprehensive studies for apparel SCs.

Moreover, the COVID-19 pandemic has proven that the traditional SC practices and mitigation strategies are insufficient for the impacts faced in a pandemic crisis (Farooq et al. 2021; Mollenkopf et al. 2020; Pourhejazy and Ashby 2021; Zhu et al. 2020). To obtain a holistic solution for the disruptions created by a pandemic, a robust decision framework is needed that addresses all the impacts on apparel SCs during a pandemic and simultaneously provide the practitioners with effective risk management strategies to ensure resilience, agility, and sustainability of future SCs. Therefore, incorporating the multi-criteria decision-making (MCDM) method can provide a holistic solution in developing the relationship between the impacts and possible mitigation strategies (Liza et al. 2022).

In this regard, to develop a decision-aid, this study explores the relationship between SCIs and their mitigation strategies during a pandemic from the lessons of the COVID-19 pandemic. Furthermore, this study also signifies the importance of studying apparel SCs by conducting a case study in the context of the apparel industry in Bangladesh, which can provide valuable insights.

The decision-making framework developed in this study addresses the following research questions (RQs):

- RQ1: What are the SCIs and consequent mitigation strategies to consider during the COVID-19 pandemic in apparel SCs?
- RQ2: How are these SCIs enlightening a prominent relationship with SC mitigation strategies?
- RQ3: Which mitigation strategies should be prioritized for overcoming the SCIs during a pandemic?

This study aims to identify the SCIs and mitigation strategies in apparel SCs amidst a pandemic from the lessons of the COVID-19 pandemic and to assess those mitigation strategies quantitatively. Furthermore, this study employs a grey-based bi-level ANP method as the model can analyze relationships between SCIs and mitigation strategies (Rajesh 2020).

The rest of the paper has been structured as follows. Section 2 presents the literature review of relevant works of the research. Following this, the methodology for the study is described in Section 3. Section 4 presents a case study and analysis of the results. Section 5 provides a discussion of the results obtained. Research implications are discussed in Section 6. Finally, this paper provides conclusions and future research scopes in Section 7.

## Literature review

SCs of different industries, such as fast-moving consumer goods, pharmaceutical, electronics, and apparel, have seen an enormous decline in the performance in logistics, procurement, manufacturing, warehousing, and all other stages that are indispensably related to the financial success of firms (Grida et al. 2020; Loske 2020; Paul and Chowdhury 2021). From the lesson of the COVID-19 pandemic, recent literature has investigated the impacts that a pandemic has on SCs globally. Notably, Swanson and Santamaria (2021) have reviewed the multi-modal impacts of the COVID-19 pandemic and discussed the ways to mitigate these disruptions. Karmaker et al. (2021) investigated the drivers of sustainable SC to reduce SC disruptions due to the pandemic in Bangladesh and identified important factors required to tackle the immediate blow on SCs due to the COVID-19 pandemic. Moreover, Hobbs (2020) investigated the challenges of SCs due to a pandemic and offered ideas on industry strategies to improve food SC resilience. Sharma et al. (2020) also noted challenges that different firms face in terms of demand–supply mismatch, technology, and the establishment of a robust SC and providing futuristic mitigation strategic proposals for SC reconstruction. The impacts of a pandemic on apparel SCs and their mitigation strategies are the prime focus of this study to develop a thoroughgoing decision-aid for the sector.

### Impacts of the COVID-19 pandemic on apparel SCs

Due to a connected market characteristic, the pandemic has hugely impacted apparel SCs due to transportation restrictions and raw materials shortages. Owing to the restriction on trade, countries with strong economies were also affected, while emerging economies took the largest hit during the COVID-19 pandemic (Caballero-Morales 2021; Coccia 2021). Countries like Bangladesh are examples of how emerging economies were significantly affected due to the economic turbulence during the COVID-19 pandemic, with an unpredictable demand pattern (Saleheen and Habib 2022). Moreover, the apparel industry’s growth as one of the most significant contributors in developing countries deteriorated drastically during this pandemic (Karmaker et al. 2021). Considering the significant impacts of the COVID-19 pandemic, sudden order cancellation has been found to cause numerous losses in apparel SCs. Additionally, the refusal of payments to the factories for material and ongoing production costs led many small and medium factories into bankruptcy, and many workers lost their jobs (Son et al. 2019; Tang 2022; Zhang et al. 2020). The dominant power of some brands was the prime reason for the fragility exposed during the pandemic in the apparel SC as this uneven distribution of power created unfair conduct by the brands (Reza and Du Plessis 2022).

Due to the distribution halt, materials became scarce, increasing the overall material and operational costs in apparel SCs, and making it more difficult for the factories to run (Paul and Chowdhury 2020). As a result, many workers were laid off (Lee et al. 2020). The majority of the factories in Bangladesh were also unable to maintain adequate health and safety measures for their workers, putting them at severe health risk (Kabir et al. 2021). On the other hand, the buyers’ requirements for safety compliance also increased, and this put pressure on small and medium companies as they lack the resources to fulfill the safety standard, resulting in the shutdown of firms (Bartik et al. 2020; Sen et al. 2020). Additionally, order fulfillment became uncertain due to strict regulations on shipments, and transportation costs also increased due to the reduced quantities (Hoek 2020; Notteboom et al. 2021). These disruptions also lowered the coordination among varied SC stages. All these impacts explored by the researchers point out the weakness in the existing SC. Researchers are still trying to identify and analyze many other SC impacts caused by a pandemic from the lessons of the COVID-19 pandemic (Paul et al. 2021). As this pandemic demonstrated the extent of disruption in SCs that any company had anticipated, there is a dire necessity to develop SC resilience and mitigation strategies to diminish these vulnerabilities (Xu et al. 2020).

### Mitigation strategies for managing SCIs

Lessening the impacts of the COVID-19 pandemic on the apparel SCs demands efficacious mitigation strategies in advance. Recent literature investigated mitigation strategies in light of the impacts faced by apparel and other SCs that made a foundation for the recovery process of the SCs. In the context of apparel SCs, Munim et al. (2022) identified that providing healthcare safety, bringing activities in-house that were earlier outsourced, and ensuring smooth delivery of existing orders are the three most significant measures for employees, suppliers, and buyers, respectively, amidst the COVID-19 pandemic. Additionally, Dutta et al. (2020) discussed the opportunities of blockchain integration in SC, considering areas such as shipping, manufacturing, automotive, and healthcare through improved discernibility to tackle the disruptions of a pandemic. Dohale et al. (2021b) identified demand uncertainty and pandemic disruption risks are the most critical risks. Based on their findings, flexibility, that is, being responsive and incorporating an agile production system to overcome crises, seems to be the most effective mitigation strategy for the apparel industry during a pandemic. Gao et al. (2021) explained the role of multi-modal transportation in minimizing time and cost through large-scale emergencies, whereas Remko (2020) mentioned research opportunities considering the quick response and alternative sources for complementing supplier segmentation. Meanwhile, the implementation of vertical and horizontal collaborations among SC partners and improved database management systems to reduce disruptions have been considered prominent strategies to overcome the impacts of the COVID-19 pandemic (Pérez-Mesa et al. 2021). Further, Leu and Masri (2021) embraced omnichannel approaches to digitizing enterprises by employing various types of technology in operations. Regarding inventory management of perishable items during the disruptions of the COVID-19 pandemic, Rana et al. (2021) modeled the impact of demand to minimize the overall cost that considers a time-dependent linear demand rate. Considering health and safety issues, Phillips et al. (2012) emphasized maintaining occupational health and safety standards for well-being during the COVID-19 pandemic. Furthermore, to overcome the impacts of the COVID-19 pandemic, Dohale et al. (2021a) prioritized visibility and transparency, flexibility, partnerships among partners, multiple sourcing, and flexible contacts as mitigation strategies. Even though there have been discussions on the mitigation strategies for apparel SCs in recent literature, more discussions are needed to formulate robust strategies to directly map the impacts of a pandemic, which can help firms develop specific actions during a pandemic.

### Applications of MCDM methods in SCs

Researchers have comprehensively used various MCDM techniques to make essential decisions in SC management. For example, Lahane and Kant (2021) prioritized risk mitigation strategies in circular SCs by using the Pythagorean Fuzzy Analytical Hierarchy Process (PF-AHP) and Pythagorean Fuzzy Vlse Kriterijumska Optimizacija Kompromisno Resenje (PF-VIKOR) methods. Additionally, fuzzy AHP was applied to analyze SC disruption drivers (Ali et al. 2021a, b). Furthermore, Chowdhury et al. (2019) prioritized the impact factors of readymade garments (RMG) SC using Interpretive Structural Modelling (ISM) and MICMAC analysis. Besides, SC collaboration barriers and strategies have been analyzed with the help of the fuzzy Best–Worst Method (BWM) and grey Decision Making Trial and Evaluation Laboratory (DEMATEL) method (Mahmud et al. 2021). Chowdhury et al. (2021a) identified the strategies for SC resilience using grey sets and the DEMATEL approach to determine the most prominent strategies, their net cause and effect, and their correlation.

ANP has already been used as an effective method separately and with other concepts in recent literature. For example, Vaidya et al. (2021) proposed an integrated ISM-ANP approach and MICMAC analysis to develop a conceptual framework and prioritize enablers for SC survivability. Moreover, Magableh and Mistarihi (2022) analyzed the impact of COVID-19 on SCs and prioritized necessary solutions considering relative importance using ANP-Technique for Order Preference by Similarity to Ideal Solution framework (ANP-TOPSIS).

This study proposed a framework called grey-based bi-level ANP as ANP can interconnect alternatives (e.g., mitigation strategies) with criteria (e.g., impacts that induce to adopt strategies) to evaluate the alternatives (Saaty and Vargas 2013). Moreover, the ambiguity and uncertainty of decision-makers can be eradicated by introducing a grey theory to the linguistic scale, which motivated us to incorporate the theory with ANP (Javanmardi et al. 2020). Moreover, ANP can simplify complex problems, quantitatively represent subjective judgment, and authorize dependence and feedback in a hierarchy more precisely than other MCDM methods (Gu et al. 2018). These advancements in grey theory and ANP are the motive behind preferring this method in our study.

## Research gaps and contributions

The COVID-19 pandemic has disrupted all aspects of SCs, which needed immediate attention from the researchers. The impact of this pandemic on apparel SCs put the logistics, supply, and demand in an unprecedented disruption. Accordingly, recent literature focused on investigating the impacts of a pandemic and developed mitigation strategies. Even though the studies provide good insights into the global SC in a generic demeanor, there has not been a justifiable contribution to holistically examining apparel SCs. Table 1 presents a list of studies focused on the impacts of apparel SCs during COVID-19 and their mitigation strategies. However, none of the studies maps a complex relationship between the impacts and specific strategies in an engaging manner. Hence, the main focus of this study is to address the gap of research in developing mitigation strategies to correlate the SCIs and ranking those mitigation strategies based on their effectiveness in minimizing SC disruptions in apparel SCs during the COVID-19 pandemic.

**Table 1:** List of recent research on SCs during the COVID- 19 pandemic

References	Apparel SCs	COVID-19	SC impacts/barriers	SC mitigation strategies
Bhandari et al. (2022); Chen et al. (2021); Perera et al. (2021); Ali et al. (2021a, b)	√		√	
Munim et al. (2022); Taqi et al. (2020)	√	√		√
Huang et al. (2021); Majumdar et al. (2021)	√		√	√
Paul et al. (2021); Ali et al. (2021a, b)	√	√	√	
Ali et al. (2021a, b); Dohale et al. (2021a, b)	√	√		√
**Current study**	√	√	√	√

Therefore, this study contributes by identifying and assessing the SCIs and mitigating strategies amidst the current pandemic, which can help practitioners in future disruptions. This study also incorporates a modified method called grey-based bi-level ANP to assess the mitigation strategies that help alleviate the disruptive SCIs. Furthermore, this decision support model can assist in deciding and implementing practical approaches to moderate SC hindrances considering apparel firms and other practical perspectives.

## Research methodology

**Fig. 1:**
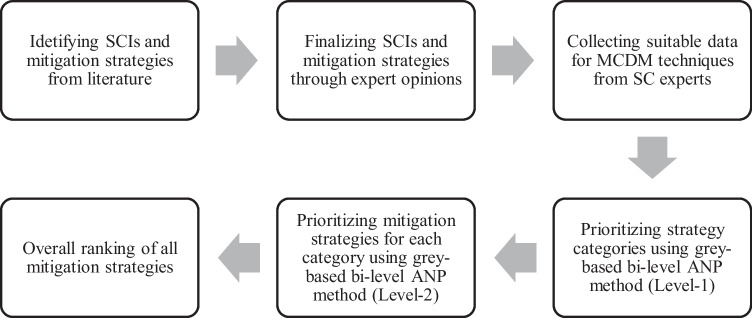
Framework for assessing mitigation strategies

The framework for this study is illustrated in Fig. 1. First, from the literature, we identified major SCIs and mitigation strategies in apparel SCs. The SCIs and mitigation strategies were then validated, expanded, and modified in apparel SCs by conducting an expert survey among SC experts. Consequently, the important relations between SCIs and mitigation strategies were determined with the help of the same SC experts. Then, the feedback was examined by using the grey-based bi-level ANP, which provides the final ranking for the strategies.

### Developing grey-based bi-level ANP model

**Fig. 2:**
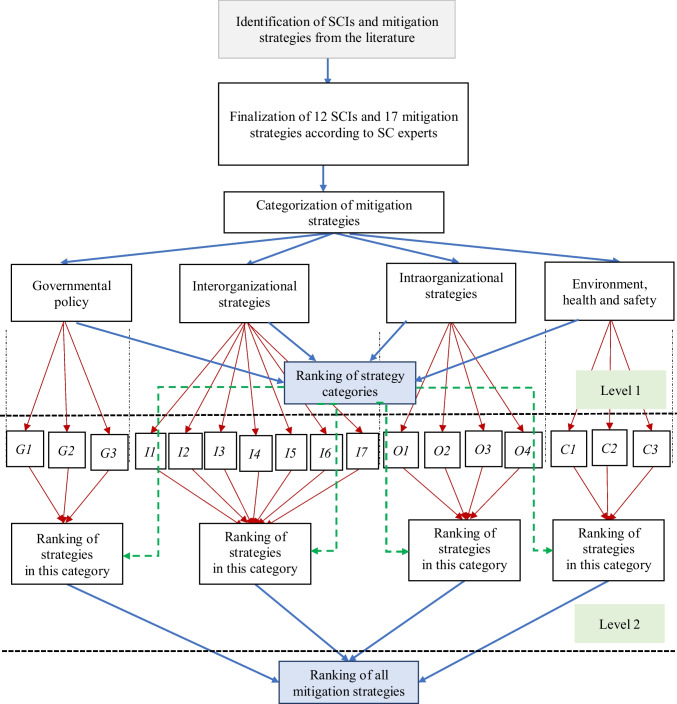
Bi-level ANP model to assess the effectiveness of strategies to mitigate SCIs

ANP is a suitable tool for establishing interrelation maps of strategies where super matrix represents those interrelations in the ANP methodology (Quezada et al. 2018). With the increasing number of strategies or any other factors, the size of the super matrix and computation complexity increases. A bi-level ANP can eradicate this problem by splitting the number of strategies (Rajesh 2020). The proposed grey-based bi-level network is presented in Fig. 2.

To avoid computational complexities in analyzing a large number of mitigation strategies, the mitigation strategies were categorized into four clusters shown in Fig. 2: Government Policy *(G1-G3)*, Interorganizational Strategies *(I1-I7)*, Intra-organizational Strategies *(O1-O4)*, and Environment, Health and Safety *(C1-C3).* In the first level, the ranking of the categories of mitigation strategies was calculated. Then, the rankings of mitigation strategies in a particular category were computed in the second level. As such, the effectiveness of the strategies was identified by utilizing the ANP method on two levels. The following steps were used during the analysis.

#### Step 1

Developing the grey relation matrices

Each expert has investigated the influence relation among the SCIs and mitigation strategies of apparel SC on a linguistic scale, as shown in Table 2. Experts’ feedback has been finalized by taking an average of the grey relation matrices. As such, five types of grey relation matrices were constructed as follows:

(i) $\left[\otimes \widetilde{{\mathrm{X}}_{\mathrm{ij}}}\right]$ indicates the influence of impacts “*i*” over “*j*”; where 1 ≤ *i* ≤ 12; 1 ≤ *j* ≤ 12.
(ii) $\left[\otimes \widetilde{{\mathrm{Y}}_{\mathrm{mij}}}\right]$ indicates the influence of categories of mitigation strategies “*i*” over “*j*” for each impact, “*m*”; where 1 ≤ *i* ≤ *4*; 1 ≤ *j* ≤ *4*; 1 ≤ *m* ≤ *12*.
(iii) $\left[\otimes \widetilde{{\mathrm{Z}}_{\mathrm{nij}}}\right]$ represents the influence of impacts “*i*” over “*j*” for each category of the mitigation strategy, “*n*”; where 1 ≤ *i* ≤ *12*; 1 ≤ *j* ≤ *12*; 1 ≤ *n* ≤ *4*.
(iv) $\left[\otimes \overline{\overline{{\mathrm{y}}_{\mathrm{mij}}}}\right]$ indicates the influence of mitigation strategies of each category “*i*” over “*j*” for each impact, “*m*”; Four sets of matrices are formed.
  $$\left[\otimes \overline{\overline{{\mathrm{Y}}_{\mathrm{mij}}^{1}}}\right]$$***;**** where* 1 ≤ *i* ≤ 3; 1 ≤ *j* ≤ 3; 1 ≤ *m* ≤ *12.*
  $$\left[\otimes \overline{\overline{{\mathrm{Y}}_{\mathrm{mij}}^{2}}}\right]$$*; where* 1 ≤ *i* ≤ 7; 1 ≤ *j* ≤ 7; 1 ≤ *m* ≤ *12.*
  $$\left[\otimes \overline{\overline{{\mathrm{Y}}_{\mathrm{mij}}^{3}}}\right]$$*; where* 1 ≤ *i* ≤ 4; 1 ≤ *j* ≤ 4; 1 ≤ *m* ≤ *12.*
  $$\left[\otimes \overline{\overline{{\mathrm{Y}}_{\mathrm{mij}}^{4}}}\right]$$*; where* 1 ≤ *i* ≤ 3; 1 ≤ *j* ≤ 3; 1 ≤ *m* ≤ *12.*
(v) $\left[\otimes \overline{\overline{{\mathrm{Z}}_{\mathrm{nij}}}}\right]$ represents the influence of impacts “*i*” over “*j*” for each mitigation strategy, “*n*”; where 1 ≤ *i* ≤ *12*; 1 ≤ *j* ≤ *12*; 1 ≤ *n* ≤ *17*.

**Table 2:** Linguistic scale using grey numbers

Linguistic scale	Grey numbers
No influence	[0,0]
Very low influence	[0,.2]
Low influence	[.2,.4]
Medium influence	[.4,.6]
High influence	[.6,.8]
Very high influence	[.8,1]

#### Step 2

Computing the crisp grey relation matrices

The crisp grey relation matrices can be obtained from average relation matrices according to the three-step modified-CFCS method (Fu et al. 2012), as shown below.

(i) Normalization:

Normalized lower limit values of the grey numbers can be computed as follows:

1 $$\underline{\otimes } \dot{{X}_{ij}}=\frac{\underline{\otimes }\widetilde{{ X}_{ij}}-{}_{j}{}^{min}\underline{\otimes }\widetilde{{ X}_{ij}}}{{\alpha }_{min}^{max}}$$

Accordingly, the values of $\underline{\otimes } \dot{{\mathrm{Y}}_{\mathrm{mij}}}$*, *$\underline{\otimes } \dot{{Z}_{nij}}$, $\underline{\otimes } {\dot{\mathrm{Y}}}_{\mathrm{mij}}^{\mathrm{g}}$, and $\underline{\otimes } \ddot{{Z}_{nij}}$ can be calculated.

Normalized upper limit values of the grey numbers can be computed as follows:

2 $$\overline{\otimes } \dot{{X}_{ij}}=\frac{\overline{\otimes }\widetilde{{ X}_{ij}}-{}_{j}{}^{min}\overline{\otimes }\widetilde{{ X}_{ij}}}{{\alpha }_{min}^{max}}$$

where,

3 $${\alpha }_{min}^{max}={}_{j}{}^{max}\overline{\otimes }\widetilde{{X}_{ij}}-{}_{j}{}^{min}\underline{\otimes }\widetilde{{ X}_{ij}}$$

$\overline{\otimes } \dot{{\mathrm{Y}}_{\mathrm{mij}}},\;\overline{\otimes } \dot{{Z}_{nij}}$, $\overline{\otimes } {\dot{\mathrm{Y}}}_{\mathrm{mij}}^{\mathrm{g}}$, $\overline{\otimes } \ddot{{Z}_{nij}}$ including ${\beta }_{min}^{max}$, ${\gamma }_{min}^{max}$, ${\dot{\beta }}_{min}^{max},\;{\dot{\gamma }}_{min}^{max}$ can be determined correspondingly.

Total Normalized Crisp Value:

4 $${X}_{ij}=\frac{\underline{\otimes } \dot{{X}_{ij}}\left(1-\underline{\otimes } \dot{{X}_{ij}}\right)+\overline{\otimes } \dot{{X}_{ij}}*\overline{\otimes } \dot{{X}_{ij}}}{1-\underline{\otimes } \dot{{X}_{ij}}+\overline{\otimes } \dot{{X}_{ij}}}$$

Accordingly, ${\mathrm{Y}}_{\mathrm{mij}}$, ${Z}_{nij}$*,*${\mathrm{Y}}_{\mathrm{mij}}^{\mathrm{g}*}$, ${\mathrm{Z}}_{\mathrm{nij}}^{*}$ can be evaluated.

(ii) Final Crisp Value:
  5 $${X}_{ij}^{*}=min\underline{\otimes }\widetilde{{ X}_{ij}}+\left({X}_{ij}*{\alpha }_{min}^{max}\right)$$
  6 $${X}^{*}=\left[{X}_{ij}^{*}\right]$$

$${Y}_{mij}^{*}$$, $${Z}_{nij}^{*},\;{Y}_{mij}^{g**}$$, $${Z}_{nij}^{**}$$ and $${Y}_{m}^{*}$$, $${Z}_{n}^{*}$$, $${Y}_{m}^{g**},\;{Z}_{n}^{**}$$ can be calculated respectively.

#### Step 3

Transforming into AHP input matrices

The values of crisp grey relation matrices have been transformed into AHP input values ranging from 5 to 1/5 as shown in Table 3 (Rajesh 2020).

**Table 3:** Transformation scaling from crisp values to AHP input values

Crisp range	AHP input
(0 0.15]	1/5
[.15 .25]	1/4
[.25 .35]	1/3
[.35 .45]	1/2
[.45 .55]	1
[.55 .65]	2
[.65 .75]	3
[.75 .85]	4
[.85 1.00)	5

7 $${X}^{*}\to \dot{{X}^{*}}$$

8 $${Y}_{m}^{*}\to \dot{{Y}_{m}^{*}}$$

9 $${Z}_{n}^{*}\to \dot{{Z}_{n}^{*}}$$

10 $${Y}_{m}^{g**}\to \dot{{Y}_{m}^{g**}}$$

11 $${Z}_{n}^{**}\to \dot{{Z}_{n}^{**}}$$

#### Step 4

Computing the Principal Normalized Eigen Vectors

We have calculated the Principal Normalized Eigen Vectors (PNEV) for each AHP input matrices as follows:

(i) ${\mathrm{e}}_{\mathrm{x}}$ denotes the PNEV value for $\dot{{X}^{*}}$ matrix.
(ii) ${\mathrm{e}}_{\mathrm{Y}}^{\mathrm{m}}$ denote the PNEV values for $\dot{{Y}_{m}^{*}}$ matrices respectively. Here, 1 ≤ *m* ≤ *12.*
(iii) ${\mathrm{e}}_{\mathrm{Z}}^{\mathrm{n}}$ denote the PNEV values for $\dot{{Z}_{n}^{*}}$ matrices. Here, 1 ≤ *n* ≤ *4.*
(iv) $${\mathrm{e}}_{\mathrm{Y}1}^{*\mathrm{m}},\;{\mathrm{e}}_{\mathrm{Y}2}^{*\mathrm{m}},\;{\mathrm{e}}_{\mathrm{Y}3}^{*\mathrm{m}},\;{\mathrm{e}}_{\mathrm{Y}4}^{*\mathrm{m}}$$ denote the PNEV values for $$\dot{{Y}_{m}^{1**}},\;\dot{{Y}_{m}^{2**},}\;\dot{{Y}_{m}^{3**},}\;\dot{{Y}_{m}^{4**}}$$ matrices, respectively. Here, 1 ≤ *m* ≤ *12.*
(v) $${\mathrm{e}}_{\mathrm{Z}}^{*\mathrm{n}}$$ denote the PNEV values for $$\dot{{Z}_{n}^{**}}$$ matrices respectively. Here, 1 ≤ *n* ≤ *17.*

#### Step 5

Establishing and analyzing the super matrices (SM) for Level-1

For Level-1, there is one super matrix, ${\mathrm{SM}}^{*}$ that consists of PNEV values of ${\mathrm{e}}_{\mathrm{x}}$*,* ${\mathrm{e}}_{\mathrm{Y}}^{\mathrm{m}}$, and ${\mathrm{e}}_{\mathrm{Z}}^{\mathrm{n}}$.

#### Step 6

Establishing and analyzing the final super matrix for Level-2

There are four super matrices for Level-2 and these are listed below.

(i) $${\mathrm{SM}}_{1}^{**}$$ consists of PNEV values $${\mathrm{e}}_{\mathrm{x}},\;{\mathrm{e}}_{\mathrm{Y}1}^{*\mathrm{m}},\;\mathrm{and}\;{\mathrm{e}}_{\mathrm{Z}}^{*\mathrm{n}}$$.
(ii) $${\mathrm{SM}}_{2}^{**}$$ consists of PNEV values $${\mathrm{e}}_{\mathrm{x}},\;{\mathrm{e}}_{\mathrm{Y}2}^{*\mathrm{m}},\;\mathrm{and}\;{\mathrm{e}}_{\mathrm{Z}}^{*\mathrm{n}}$$_**.**_
(iii) $${\mathrm{SM}}_{3}^{**}$$ consists of PNEV values $${\mathrm{e}}_{\mathrm{x}},\;{\mathrm{e}}_{\mathrm{Y}3}^{*\mathrm{m}},\;\mathrm{and}\;{\mathrm{e}}_{\mathrm{Z}}^{*\mathrm{n}}$$.
(iv) $${\mathrm{SM}}_{4}^{**}$$ consists of PNEV values $${\mathrm{e}}_{\mathrm{x}},\;{\mathrm{e}}_{\mathrm{Y}4}^{*\mathrm{m}},\;\mathrm{and}\;{\mathrm{e}}_{\mathrm{Z}}^{*\mathrm{n}}$$.

From the super matrix of Level-1, the categories can be ranked. Then from Level- 2, mitigation strategies of each category can be ranked with the help of local weights. We can compute the global weights of mitigation strategies from these two levels. Finally, the rankings of the mitigation strategies can be determined based on the global weights.

## Case study and result analysis

To evaluate the effectiveness of the proposed model, a case study is conducted on an apparel manufacturing company to identify SCIs during the COVID-19 pandemic and their mitigation strategies. Due to the confidentiality agreement, the company’s name is not disclosed. The company is one of Bangladesh’s largest export-oriented companies of different clothing types, such as sweaters, shirts, socks, and various accessories. They operate on an order basis and mainly export products to various countries. The SCIs have been higher for this company as a global manufacturing company than most local companies due to this pandemic. Hence, the company’s portfolio fits appropriately for the case study to provide meaningful insights into the impacts that apparel SCs may face during a pandemic and strategies to mitigate them. The proposed methodology was applied to provide qualitative and quantitative mitigation strategies to reduce the vulnerability of SCIs.

### Data collection

Selected SCIs and mitigation strategies for apparel SCs have been accumulated considering relevant literature and expert’s opinion in this study. Initially, eight SCIs were identified by studying the relevant literature. Considering the recent studies on apparel SCs, the impacts of the COVID-19 pandemic were examined while selecting the SCIs. Next, the list of SCIs was shared with nine industry experts of the above-mentioned company via email. These experts were chosen for their extensive industry knowledge and eagerness to participate in the review process. The experts’ credentials are mentioned in Table 11 of Appendix. Along with the list from the existing literature, experts also suggested four new SCIs in the context of apparel SCs. In total, 12 SCIs were identified in this study with the help of existing literature and surveys from experts.

Similarly, a list containing eight mitigation strategies, identified from the literature, was shared with the same experts for their feedback. Experts suggested nine new mitigation strategies. Subsequently, a list of 17 mitigation strategies was finalized based on the literature review and experts’ opinions. These SCIs and mitigation strategies are shown in Tables 4 and 5, respectively. The methodologies described in the previous section are applied to this case study in a step-by-step procedure.

**Table 4:** List of SCIs

Notation	Name of the impact	Description	Source (LR = Literature Review; EO = Expert Opinion)
SCI1	Decelerated demand	A drastic fall in the distribution frequency of apparel products led to a downward trend in the consumer buying pattern	EO
SCI2	Increased material and operational costs	Unavailability and inaccessibility of raw materials and subsequent lockdowns increased material and operational costs	LR (Paul and Chowdhury 2020)
SCI3	Undesirable increased lead time	Difficulty in distribution due to transportation restrictions led to increased lead time for the product to reach the customers	EO
SCI4	Inadequate health and safety measures	Working during the COVID-19 pandemic with insufficient safety measures has put workers and employees of firms at major health and safety risks	EO + LR (Kabir et al. 2021)
SCI5	Sudden surge in the unemployment rate	Immediate closing down of factories led to the unemployment of millions of factory workers	LR (Lee et al. 2020)
SCI6	Uncertainty in overseas order fulfillment	Strict regulations on international borders raised uncertainty about overseas shipment during the COVID-19 pandemic	LR (Notteboom et al. 2021)
SCI7	Increased pressure to improve safety compliance and standards	Pressure from foreign buyers for immediate improvement of safety compliances and standards led firms to halt their operations temporarily	EO
SCI8	Cancellation of orders and delayed payment	Escalated cancellation of orders from buyers and reluctance to compensate the cost of materials and production led to significant financial risk in apparel SCs	EO + LR (Majumdar et al. 2020)
SCI9	Increased transportation cost	A decrease in the volume of activities due to the COVID-19 pandemic led to increased delivery charges by the distributors	LR (Hoek 2020)
SCI10	Salary cut of employees	A decrease in the volume of operation and revenue forced companies to reduce the salary of their employees	EO
SCI11	Shut down of small firms and retailers	Critically affected areas by the COVID-19 pandemic made small firms and retailers close down their facilities	EO + LR (Bartik et al. 2020)
SCI12	Lack of coordination among different stages of SC	Increased vulnerability in SC during the COVID-19 pandemic dropped the coordination among SC stages	LR (McMaster et al. 2020)

**Table 5:** List of mitigation strategies

Category	Notation	Mitigation strategy	Source (LR = Literature Review; EO = Expert Opinion)
**Governmental policy**	**G1**	Incentivizing apparel SCs through formulation and implementation of different policies and financial aid plans	EO
	**G2**	Easing customs and regulatory processes in cross-national transactions amidst the COVID-19 pandemic	EO
	**G3**	Rescheduling loan installment span for critically affected apparel SCs to endure the effect of the COVID-19 pandemic	EO
**Interorganizational strategies**	**I1**	Implementing a multi-modal transportation system to reduce the uncertainty of distribution among different stages	LR (Gao et al. 2021)
	**I2**	Developing a sustainable and resilient relationship with local sources to alleviate the risk of international supply during crises	LR (Remko 2020)
	**I3**	Developing operational and tactical policies to tackle the downward slope of demand and Bullwhip effects	LR (Bamakan et al. 2021)
	**I4**	Incorporating vertical and horizontal collaboration among different SC partners for holistic SC practice	EO + LR (Pérez-Mesa et al. 2021)
	**I5**	Building action plans to stop the propagation of disturbance caused by the ripple effect	LR (Ivanov and Dolgui 2020)
	**I6**	Establishing advanced data management system and analytics for precise prediction of net requirement and information of distribution to reduce disruption	EO
	**I7**	Redesigning SCs to enable a transparent circular economy through blockchain technology	LR (Dutta et al. 2020)
**Intra-organizational strategies**	**O1**	Constructing omnichannel retailer distribution within apparel SCs to increase responsiveness	EO + LR (Leu and Masri 2021)
	**O2**	Adopting a holistic marketing approach for increased corporate social responsibilities attracts alternate buyers and markets	EO
	**O3**	Adopting an agile and flexible management system to enrich inventory and resource capacity plan for faster response to the COVID-19 pandemic	EO
	**O4**	Introducing flexible payment methods in apparel SCs to ease transactions during the COVID-19 pandemic	EO
**Environment, health, and Safety**	**C1**	Adopting necessary occupational and behavioral change (proper usage of personal protective kits, following pandemic protocols) during the COVID-19 pandemic	EO
	**C2**	Introducing state-of-the-art occupational safety standards and health measures for increased sustainability and well-being in the COVID-19 pandemic	EO + LR (Phillips et al. 2012)
	**C3**	Conducting training programs for workers regarding workplace health and safety issues focusing on the pandemic situation	EO

### Grey-based bi-level ANP

#### Step 1

Five sets of grey relations matrices were constructed from the surveys of the same experts mentioned in Section 4.1 using the linguistic scale shown in Table 2. The matrix $\left[\otimes \widetilde{{\mathrm{X}}_{\mathrm{ij}}}\right]$ is shown in Table 6. Similarly, other grey relation matrices were constructed.

**Table 6:** Grey matrix of the relative importance of impacts in SC

	SCI1	SCI2	SCI3	SCI4	SCI5	SCI6	SCI7	SCI8	SCI9	SCI10	SCI11	SCI12
SCI1	[0,0]	[.8,1]	[.4,.6]	[.2,.4]	[.6,.8]	[.4,.6]	[.2,.4]	[.8,1]	[.8,1]	[.8,1]	[.6,.8]	[.2,.4]
SCI2	[0, .2]	[0,0]	[.2,.4]	[0,.2]	[.4,.6]	[.2,.4]	[.2,.4]	[.4,.6]	[.4,.6]	[.6,.8]	[.6,.8]	[.2,.4]
SCI3	[.2,.4]	[.6,.8]	[0,0]	[0,.2]	[.2,.4]	[.2,.4]	[0,.2]	[.8,1]	[.8,1]	[.4,.6]	[.4,.6]	[.4,.6]
SCI4	[.4,.6]	[.4,.6]	[.4,.6]	[0,0]	[.4,.6]	[0,.2]	[.8,1]	[.6,.8]	[.6,.8]	[.4,.6]	[.4,.6]	[.2,.4]
SCI5	[0,.2]	[.2,.4]	[0,.2]	[0,.2]	[0,0]	[0,.2]	[.2,.4]	[.2,.4]	[.2,.4]	[0,.2]	[.4,.6]	[.2,.4]
SCI6	[.2,.4]	[.6,.8]	[.4,.6]	[.4,.6]	[.8,1]	[0,0]	[.2,.4]	[.6,.8]	[.6,.8]	[.6,.8]	[.4,.6]	[.2,.4]
SCI7	[.2,.4]	[.6,.8]	[.4,.6]	[.6,.8]	[.8,1]	[.4,.6]	[0,0]	[.4,.6]	[.2,.4]	[.4,.6]	[.6,.8]	[.2,.4]
SCI8	[.4,.6]	[.6,.8]	[.6,.8]	[.4,.6]	[.6,.8]	[.4,.6]	[.4,.6]	[0,0]	[.6,.8]	[.8,1]	[.6,.8]	[.4,.6]
SCI9	[.2,.4]	[.2,.4]	[0,.2]	[.2,.4]	[.4,.6]	[0,.2]	[0,.2]	[0,.2]	[0,0]	[.6,.8]	[.4,.6]	[.2,.4]
SCI10	[.2,.4]	[.4,.6]	[.2,.4]	[.4,.6]	[.4,.6]	[.4,.6]	[.2,.4]	[.2,.4]	[.4,.6]	[0,0]	[.2,.4]	[.4,.6]
SCI11	[.4,.6]	[.8,1]	[.8,1]	[.6,.8]	[.8,1]	[.6,.8]	[.6,.8]	[.6,.8]	[.8,1]	[.8,1]	[0,0]	[.2,.4]
SCI12	[.2,.4]	[.4,.6]	[.4,.6]	[.2,.4]	[.4,.6]	[0,.2]	[.2,.4]	[.2,.4]	[.4,.6]	[.4,.6]	[0,.2]	[0,0]

#### Step 2

The crisp relation matrices are obtained from grey relation matrices using Eqs. (1)–(6). The crisp matrix, $${X}^{*}$$ is shown in Table 7. Similarly, $${Y}_{m}^{*}$$*,* $${Z}_{n}^{*},\;{Y}_{m}^{g**}$$, and $${Z}_{n}^{**}$$ were obtained.

**Table 7:** Crisp matrix of the relative importance of impacts in SC

	SCI1	SCI2	SCI3	SCI4	SCI5	SCI6	SCI7	SCI8	SCI9	SCI10	SCI11	SCI12
SCI1	0	0.97	0.5	0.28	0.73	0.52	0.27	0.97	0.97	0.97	0.76	0.3
SCI2	0.05	0	0.27	0.04	0.5	0.28	0.27	0.5	0.5	0.73	0.76	0.3
SCI3	0.3	0.73	0	0.04	0.27	0.28	0.03	0.97	0.97	0.5	0.52	0.55
SCI4	0.55	0.5	0.5	0	0.5	0.04	0.97	0.73	0.73	0.5	0.52	0.3
SCI5	0.05	0.27	0.03	0.04	0	0.04	0.27	0.27	0.27	0.03	0.52	0.3
SCI6	0.3	0.73	0.5	0.52	0.97	0	0.27	0.73	0.73	0.73	0.52	0.3
SCI7	0.3	0.73	0.5	0.76	0.97	0.52	0	0.5	0.27	0.5	0.76	0.3
SCI8	0.55	0.73	0.73	0.52	0.73	0.52	0.5	0	0.73	0.97	0.76	0.55
SCI9	0.3	0.27	0.03	0.28	0.5	0.04	0.03	0.03	0	0.73	0.52	0.3
SCI10	0.3	0.5	0.27	0.52	0.5	0.52	0.27	0.27	0.5	0	0.28	0.55
SCI11	0.55	0.97	0.97	0.76	0.97	0.76	0.73	0.73	0.97	0.97	0	0.3
SCI12	0.3	0.5	0.5	0.28	0.5	0.04	0.27	0.27	0.5	0.5	0.04	0

#### Step 3

The crisp input matrices were transformed into AHP input values with ranges shown in Table 2. The AHP input matrix, $$\dot{{X}^{*}}$$ is shown in Table 8 and the other AHP matrices, $$\dot{{Y}_{n}^{*}, }\;\dot{{Z}_{n}^{*}}\dot{{Y}_{m}^{g**}}$$, and $$\dot{{Z}_{n}^{**}}$$ were developed similarly.

**Table 8:** AHP input matrix of the relative importance of impacts in SC

	*SCI1*	*SCI2*	*SCI3*	*SCI4*	*SCI5*	*SCI6*	*SCI7*	*SCI8*	*SCI9*	*SCI10*	*SCI11*	*SCI12*
*SCI1*	0	5	1	1/3	3	1	1/3	5	5	5	4	1/3
*SCI2*	1/5	0	1/3	1/5	1	1/3	1/3	1	1	3	4	1/3
*SCI3*	1/3	3	0	1/5	1/3	1/3	1/5	5	5	1	1	2
*SCI4*	2	1	1	0	1	1/5	5	3	3	1	1	1/3
*SCI5*	1/5	1/3	1/5	1/5	0	1/5	1/3	1/3	1/3	1/5	1	1/3
*SCI6*	1/3	3	1	1	5	0	1/3	3	3	3	1	1/3
*SCI7*	1/3	3	1	4	5	1	0	1	1/3	1	4	1/3
*SCI8*	2	3	3	1	3	1	1	0	3	5	4	2
*SCI9*	1/3	1/3	1/5	1/3	1	1/5	1/5	1/5	0	3	1	1/3
*SCI10*	1/3	1	1/3	1	1	1	1/3	1/3	1	0	1/3	2
*SCI11*	2	5	5	4	5	4	3	3	5	5	0	1/3
*SCI12*	1/3	1	1	1/3	1	1/5	1/3	1/3	1	1	1/5	0

#### Step 4

The principal normalized Eigenvectors*,* $${\mathrm{e}}_{\mathrm{X}},$$ $${\mathrm{e}}_{\mathrm{Y}}^{\mathrm{m}},\;{\mathrm{e}}_{\mathrm{Z}}^{\mathrm{n}}$$  $${\mathrm{e}}_{\mathrm{Y}1}^{*\mathrm{m}},\;{\mathrm{e}}_{\mathrm{Y}2}^{*\mathrm{m}},\;{\mathrm{ e}}_{\mathrm{Y}3}^{*\mathrm{m}},\;{\mathrm{e}}_{\mathrm{Y}4}^{*\mathrm{m}}$$*,* and $${\mathrm{e}}_{\mathrm{Z}}^{*\mathrm{n}}$$ of AHP matrices ($$\dot{{X}^{*},}\;\dot{{Y}_{n}^{*}, }\;\dot{{Z}_{n}^{*}}\dot{{Y}_{m}^{g**}}$$, and $$\dot{{Z}_{n}^{**}}$$) were calculated.

#### Step 5

The super matrix of Level-1, ${SM}^{*}$ constructed with the values of ${e}_{X},\;{e}_{Y}^{m}$, and ${e}_{Z}^{n}$ is shown in Table 8. The weights of each criterion obtained from this super matrix are shown in Table 9.

**Table 9:** ANP super matrix, ${SM}^{*}$ of level-1

			Criteria													Alternatives			
		**Select**	**SCI1**	**SCI2**	**SCI3**	**SCI4**	**SCI5**	**SCI6**	**SCI7**	**SCI8**	**SCI9**	**SCI10**	**SCI11**	**SCI12**	***G***		***I***	***O***	***C***
	**Select**	1	0	0	0	0	0	0	0	0	0	0	0	0	0		0	0	0
Criteria	**SCI1**	0.112385	1	0	0	0	0	0	0	0	0	0	0	0	0.043		0.182	0.129	0.03
	**SCI2**	0.048133	0	1	0	0	0	0	0	0	0	0	0	0	0.055		0.035	0.104	0.02
	**SCI3**	0.079706	0	0	1	0	0	0	0	0	0	0	0	0	0.042		0.117	0.085	0.02
	**SCI4**	0.100815	0	0	0	1	0	0	0	0	0	0	0	0	0.032		0.045	0.056	0.25
	**SCI5**	0.019693	0	0	0	0	1	0	0	0	0	0	0	0	0.085		0.025	0.056	0.05
	**SCI6**	0.080082	0	0	0	0	0	1	0	0	0	0	0	0	0.12		0.138	0.146	0.02
	**SCI7**	0.09645	0	0	0	0	0	0	1	0	0	0	0	0	0.105		0.025	0.034	0.3
	**SCI8**	0.138096	0	0	0	0	0	0	0	1	0	0	0	0	0.082		0.142	0.133	0.02
	**SCI9**	0.030857	0	0	0	0	0	0	0	0	1	0	0	0	0.029		0.052	0.042	0.02
	**SCI10**	0.05433	0	0	0	0	0	0	0	0	0	1	0	0	0.082		0.046	0.045	0.05
	**SCI11**	0.209417	0	0	0	0	0	0	0	0	0	0	1	0	0.194		0.097	0.134	0.22
	**SCI12**	0.030037	0	0	0	0	0	0	0	0	0	0	0	1	0.133		0.097	0.037	0.02
Alternatives	***G***	0	0.411	0.472	0.32	0.137	0.202	0.53	0.08	0.168	0.223	0.193	0.409	0.48	1		0	0	0
	***I***	0	0.235	0.453	0.4	0.208	0.552	0.22	0.23	0.645	0.409	0.582	0.399	0.318	0		1	0	0
	***O***	0	0.185	0.037	0.24	0.223	0.13	0.15	0.12	0.094	0.288	0.128	0.07	0.095	0		0	1	0
	***C***	0	0.169	0.037	0.04	0.431	0.117	0.09	0.57	0.093	0.079	0.097	0.122	0.107	0		0	0	1

#### Step 6

Subsequently, four separate super matrices, ${SM}_{1}^{*},\;{SM}_{2}^{*},\;{SM}_{3}^{*}$, and ${SM}_{4}^{*}$ were constructed in Level-2. Local weights of each mitigation strategy shown in Table 9 were calculated. Finally, the global weights of the mitigation strategies shown in Table 10 were calculated using the categories’ weights and local weights of the mitigation strategies. With these global weights, the final rankings of the mitigation strategies were identified.

**Table 10:** Global weights and final rankings of mitigation strategies with the help of grey-based bi-level ANP method

Category name	Category weights	Mitigation strategies	Local weights of mitigation strategies	Global weights of mitigation strategies	Final ranking
Government policy	0.303	G1	0.40615	0.12306	1
		G2	0.248	0.07514	4
		G3	0.34301	0.10393	2
Interorganizational Strategies	0.378	I1	0.12674	0.04791	12
		I2	0.12848	0.04856	11
		I3	0.1513	0.05719	7
		I4	0.14799	0.05594	8
		I5	0.12992	0.04911	9
		I6	0.1865	0.0705	5
		I7	0.12924	0.04885	10
Intra-organizational Strategies	0.137	O1	0.33165	0.04544	13
		O2	0.32634	0.04471	14
		O3	0.19015	0.02605	16
		O4	0.1518	0.0208	17
Environment, health, and safety	0.182	C1	0.33857	0.06162	6
		C2	0.22981	0.04183	15
		C3	0.43162	0.07856	3

## Discussion of findings

With the final rankings obtained using the grey-based bi-level ANP method, decision-makers of an apparel firm can take comprehensive steps to recover from the disruptions in SC that may be affected by a pandemic. The effectiveness of the mitigation strategies can also be analyzed with the help of their ranking orders.

The findings from the case study suggest that to mitigate the SCIs, the incorporation of the most important mitigation strategy, “Incentivizing apparel SCs through formulation and implementation of different policies and financial aid plans *(G1)*” can be a great start. Sequentially, other mitigation strategies can be followed. Implementing government policies and financial aid can help firms sustain, especially during a crisis. Formulating and implementing government rules assist firms in tackling several SCIs, such as “Increased material and operational costs *(SCI2)*,” “The sudden surge in the unemployment rate *(SCI5)*,” “Cancellation of orders and delayed payment *(SCI8)*,” “Increased transportation cost *(SCI9)*,” “Salary cut of employees *(SCI10)*,” and “Shut down of small firms and retailers *(SCI11)*.” It can also partially mitigate “Uncertainty in overseas order fulfillment *(SCI6)*.” As these SCIs lead to a sudden increase in costs, travel restrictions, decrease, and the cancellation of orders and payments, their severity can be mitigated with government incentives and policies. Thus, *G1* was prioritized analytically. Hence, it ranked first in the global ranking of mitigation strategies, as shown in Table 10, indicating its importance in mitigating the SCIs during the pandemic.

“Rescheduling loan installment span for critically affected apparel SCs to endure the effect of the COVID-19 pandemic *(G3)*,” ranked second in our case study, can further enhance the capability of the apparel company to endure the SCIs discussed above caused by the COVID-19 pandemic. “Conducting training programs for workers regarding workplace health and safety issues focusing on the pandemic situation *(C3)*,” is one step closer to implementing safety protocols and improving safety standards. Thus, this strategy can help maintain adequate safety measures and improve safety compliance and standards (to tackle “Inadequate health and safety measures (*SCI4)*” and “Increased pressure to improve safety compliance and standards *(SCI7)*.” This strategy can also help prevent the closing of small companies which were not getting enough orders due to safety issues.

“Establishing advanced data management system and analytics for precise prediction of net requirement and information of distribution to reduce disruption *(I6)*”, ranked fifth in listed mitigation strategies, can help the companies to predict the net requirements and identify any SC disruptions. Thus, the SCIs can be identified before their occurrences, and companies can act accordingly. On the other hand, “Adopting necessary occupational and behavioral change (proper usage of personal protective kits, following pandemic protocols) during the COVID-19 pandemic *(C1)*” suggests installing certain behavioral changes to the employee to be cautious and eager to maintain the safety precautions. Therefore, the safety conditions of the factories can be improved. Moreover, with mitigation strategies like “Developing operational and tactical policies to tackle the downward slope of demand and Bullwhip effects *(I3)*” and “Incorporating vertical and horizontal collaboration among different SC partners for holistic SC practice *(I4)*,” the firms can tackle the downward slope of demand and bullwhip effects. Since the downward slope of demand during the pandemic reduces the integration among different stages of SCs, which induces the bullwhip effect, every stage of SCs faces SCIs like *SCI2* and *SCI9*. Hence, increasing collaboration among SC partners can reduce the bullwhip effects, and subsequently, formulating tactical plans can help reduce the overall operational cost.

“Developing a sustainable and resilient relationship with local sources to alleviate the risk of international supply under crises *(I2)*” can further help the industry tackle material scarcity due to the restrictions on international borders. Consequently, material costs may not increase rapidly because of the availability of local sources. On the other hand, “Implementing a multi-modal transportation system to reduce the uncertainty of distribution among different stages *(I1)*” can help reduce the uncertainty in order fulfillment and lead time; thus, it can reduce the impacts of *SCI6* and *SCI3*. Simultaneously, “Adopting a holistic marketing approach for increased corporate social responsibilities attracts alternate buyers and markets *(O2)*” can attract new buyers and mitigate the impacts caused by the monopoly of a few buyers.

From the discussions above, it can be stated that every mitigation strategy can tackle several impacts on apparel SCs during a pandemic. Nevertheless, implementing the mitigation strategies identified in this study can assist in the recovery of apparel SCs tacking the SCIs caused by future pandemics and unprecedented events. These mitigation strategies can also help the SCs of other sectors formulate plans for future uncertainty and disruptions.

## Research implications

This section highlights the implications of the framework formulated in this study in the disruptive crisis of apparel SCs during a global pandemic like COVID-19. The implications have been presented from a theoretical and a practical perspective to demonstrate the framework’s usability as an immediate response measure for the recovery of the financial and operational breakdown of apparel SCs.

### Theoretical implications

This study addresses inconsistency and disruption in SC due to a calamity, such as the COVID-19 pandemic, by exploring major SCIs. Additionally, the SCIs discussed in this study provide a holistic view of understanding critical and vulnerable aspects of SCs during a pandemic. The findings reveal that the operational capability of SCs is greatly affected during a pandemic. Additionally, resource shortages become a significant concern during an emergency, which results in increased material and operations costs along with delayed throughput (Paul and Chowdhury 2020; Majumdar et al. 2020). Moreover, the coordination among SC partners is greatly affected due to disruptions in performance which impose uncertainty in the operational output of SCs, employment, and financial sustainability of firms.

From a theoretical perspective, this study reveals that developing robustness-based multidisciplinary mitigation strategies can address SCIs caused by a pandemic in all possible stages of the apparel SCs. The results of this study show that legislative effort can play a more prominent role in addressing SCIs during an emergency than technological changes. While financial aid planning from policymakers can be instrumental, leveraging health and safety protocols are also important in maintaining firms’ occupational sustainability during emergencies (Pamucar et al. 2022). Furthermore, the study emphasizes a collaborative approach (i.e., data management system and vertical collaboration) among partners to increase their capacity to absorb ripple effect and demand uncertainty (Bamakan et al. 2021; Pérez-Mesa et al. 2021). Theoretically, inter-organizational collaboration is predominant in accomplishing a broader range of mitigation strategies in an emergency. While intra-organizational strategies can only help SCs to survive during an emergency, a collaborative approach can ensure the sustainability and resilience of SCs.

Finally, to quantify the mitigation strategies for the probable impacts of a pandemic in a complex environment, the study uses an analytical approach, grey-based bi-level ANP, as an aid to apparel SCs in a disruptive environment. This approach maps the relationship between SCIs and mitigation strategies for apparel SCs and recognizes strategies with more priority, which reveals the predominance of policy-related and collaborative strategies to address SCIs.

### Managerial implications

This study aimed to explore and identify possible pandemic-related SCIs on the apparel SCs and develop a decision-making framework that provides a priority-based comprehensive set of mitigation strategies for those impacts. The study findings have several managerial implications for apparel practitioners during an emergency, which can significantly help managers and policymakers endure remedial action and sustain SC resilience.

With regard to financial aid planning, it is evident that extensive financial support from SC practitioners and the government can strengthen the apparel SC in the recovery process (Karmaker et al. 2021). To elaborate, the results reveal that policymakers should emphasize formulating and implementing financial aid plans during an emergency. A holistic approach can include a set of aid packages for economic recovery considering a firm’s size, working rate, and employee percentage (Islam et al. 2020a, b). Further, the impacts of an emergency can disrupt the regular payment of loan installments due to the economic instability of firms. Policymakers can formulate necessary steps to reschedule the loan installment span for critically affected apparel SCs.

Moreover, it is indispensable for management to impose safety rules and regulations considering the nature of the emergency. Managers can design appropriate training programs and campaigns to ease the process (Ferguson and Drake 2021). It is also vital that practitioners pay more attention to increasing vertical and horizontal collaborations with SC partners and formulate collaborative action plans to respond quickly and adapt to an emergency business environment (Ivanov and Dolgui 2020; Bamakan et al. 2021). Nevertheless, practitioners should constantly look for innovations to make operations and management flexible. To increase flexibility, designing omnichannel networks, selecting multiple suppliers, and creating flexible payment systems are important (Leu and Masri 2021; Chowdhury et al. 2021a).

Finally, the proposed methodology offers policymakers a multi-dimensional set of strategic plans based on their importance. Analyzing mitigation strategies and their relationships ensures a robust selection of mitigation strategies. It also strengthens the collaboration with partners, contributes to social responsibilities, and improves the currently disrupted status quo of the global apparel SC.

## Conclusions and future research scopes

With the elevating negative impacts of the COVID-19 pandemic, the global economy has seen a significant depression affecting millions of lives. The apparel SC is one of the largest contributors to the global economy and has supposedly been affected to an unforeseeable scale by the pandemic. The pandemic taught practitioners that apparel SCs needed a proactive approach that could explore, identify, and measure the severity of major SCIs that induced vulnerabilities in operational performances during a pandemic and alleviate them through a systematic strategic framework. This research contributes to the immediate need for a systematic framework by finding the crucial SCIs and developing a ranked-based model for strategies that can mitigate the severity of the SCIs. A grey-based bi-level ANP method proposed in the research quantifies the empirical findings of mitigation strategies to quantitative analysis. This approach helps policymakers prioritize the strategies while implementing them based on their rankings. The bi-level ANP method extends its advantage by developing a category-wise ranking in a complex mitigation strategy listing. The framework reveals the necessity of legislative and collaborative effort and increasing organizational capacity to address disruptions in an emergency. This framework helps in managerial decision-making within an organization and expands beyond the organization for better collaboration amongst partners, regulators, and other stakeholders. The study reflects its effectiveness in building flexibility across all the stages of the apparel SCs. To conclude, the research can be helpful for managers to manage all the SC vulnerabilities during a pandemic by mitigating severe impacts with the important strategies sought by the current market.

The study has some limitations that could be addressed to expand the research domain further. One of the major drawbacks of the study is providing a framework for SC mitigation strategies at the strategic level. In practice, a function-focused analysis could prioritize tactical and operational strategies. The advantage of such a study is that it provides a micro-analysis of procurement, manufacturing, inventory management, and distribution. The number of impacts can be increased through more exploration for a more robust solution for the apparel SCs. Similarly, a few more mitigation strategies can be considered to mitigate the impending impacts on a larger scale. There might be biases in assigning ratings to different pair-wise comparisons that apparel company managers and other SC practitioners provided. The approach eliminated cluster priorities to avoid further complexities in the analysis, which could have been addressed.

The study’s limitations suggest that a similar analysis can be carried out at the functional level, such as logistics, manufacturing, and inventory management. The study’s findings can be used in other fields of SC, notably in the pharmaceutical, fast-moving consumer goods, and automobile industries. Comparing another approach (fuzzy theory) with the grey-based bi-level ANP method may verify the accuracy of the current findings. Furthermore, some of the behavioral attributes (e.g., strategic behavior of employers, ethical concern, risk attitude, and consumer behavior) can be considered for developing a better framework.

## Data Availability

The authors confirm that the analysis data of this study are available within the article and its appendices.

## Appendix

**Table 11:** The profiles of experts

Occupations	Expert Name	Position	Experience (Years)
Industry Experts	Expert 1	Senior Manager, SC Department	10
	Expert 2	Deputy General Manager, Planning Department	16
	Expert 3	Senior Manager, SC Department	13
	Expert 4	Senior Manager, Planning Department	10
	Expert 5	Manager, SC Department	12
	Expert 6	Manager, Planning Department	10
	Expert 7	Senior Executive, Procurement	7
	Expert 8	Senior Manager, Production Planning	12
	Expert 9	Manager, Merchandiser	11
